# Perceived school bullying and psychotic-like experiences in sexual minority adolescents: the mediating and moderating roles of resilience

**DOI:** 10.1186/s13034-024-00747-7

**Published:** 2024-05-16

**Authors:** Dongfang Wang, Xiao-Yan Chen, Andrew Scherffius, Zhijun Yu, Xuan Wang, Meng Sun, Fang Fan

**Affiliations:** 1 School of Psychology, Centre for Studies of Psychological Applications, Guangdong Key Laboratory of Mental Health and Cognitive Science, Ministry of Education Key Laboratory of Brain Cognition and Educational Science, Guangdong Emergency Response Technology Research Center for Psychological Assistance in Emergencies, South China Normal University, Guangzhou, China; 2 School of Psychology, Fujian Normal University, Fuzhou, China; 3 Perelman School of Medicine, University of Pennsylvania, Philadelphia, PA USA; 4 Department of Social Psychiatry, The Affiliated Brain Hospital of Guangzhou Medical University (Guangzhou Huiai Hospital), Guangzhou, China; 5 School of Psychology, South China Normal University, Shipai Road, Guangzhou, China

**Keywords:** Perceived school bullying, Resilience, Psychotic-like experience, Sexual minority, Mediation, Moderation

## Abstract

**Aims:**

This two-wave, longitudinal study aimed to examine the potential moderating and mediating effects of resilience on the association between perceived school bullying and psychotic-like experiences among Chinese sexual minority adolescents.

**Methods:**

A total of 4192 senior high students were included and 984 (23.5%) of them were identified as a sexual minority (mean age = 16.68 years, SD = 0.71). Participants completed two online surveys during April 21 to May 12, 2021 and December 17 to 26, 2021, respectively, as well as completed self-report measures of sample characteristics, perceived school bullying, resilience, and psychotic-like experiences (including two dimensions: delusional experiences and hallucinatory experiences).

**Results:**

Perceived school bullying and resilience were associated with psychotic-like experiences in sexual minority adolescents. Resilience mediated the relationship between perceived school bullying and subsequent psychotic-like experiences (b = 0.03, 95% CI = 0.01 ~ 0.04)/ delusional experiences (b = 0.03, 95% CI = 0.01 ~ 0.04)/ hallucinatory experiences (b = 0.02, 95% CI = 0.01 ~ 0.03). Additionally, resilience only moderated the associations of perceived school bullying with hallucinatory experiences (b = −0.06, 95% CI = −0.12 ~ −0.01).

**Conclusions:**

These findings indicated that resilience plays a crucial role in mediating or moderating the relationship between perceived school bullying and psychotic-like experiences. Assessing and reducing school bullying, as well as promoting resilience, may have important clinical implications for reducing the risk of psychotic-like experiences in sexual minority adolescents.

## Introduction

Psychotic-like experiences (PLEs) are defined as experiences that resemble the positive symptoms of psychosis in the absence of a full-blown psychotic disorder [1]. A growing body of research has focused on PLEs in children and adolescents, because PLEs at an early age often predict the later onset of serious psychopathology [2–4]. Indeed, PLEs are common in youth and adolescent samples, with 17% of children 9 ~ 12 and 7.5% of adolescents aged 13 ~ 18 years having PLEs [5], which was significantly higher than in the adult population (~ 5%) [6, 7]. In China, one survey of 5427 adolescents showed that 95.7% of adolescents had had at least one PLE during their lifetime, while 17.2% continued to experience frequent PLEs [8]. More recently, a large sample study showed that the prevalence of monthly PLEs was 15.4% among adolescents in urban China [9]. Given the relatively high prevalence of PLEs among adolescents and the strong predictability of psychological symptoms, it is necessary to explore their influencing factors in order to reduce their rates.

A recent meta-analysis of 23 studies from 8 countries suggests that the proportion of sexual minority adolescents in the studies ranged from 2.3 to 12% [10]. One recent study showed that 4.1% of Chinese adolescents self-reported as sexual minorities and 17.3% were unsure [11]. Compared to other demographic groups, sexual minorities may experience a greater frequency of PLEs. Based on the minority stress model [12], sexual minorities tend to suffer from excess stress (e.g., stigmatized social status, discrimination, and violence), which may lead to mental health issues, such as depression [13], anxiety [14], suicidality [15], and psychotic symptoms [16]. The association between sexual minority status and PLEs appears inconclusive. For instance, one study among the general population in England showed that non-heterosexual orientation was associated with greater risk of PLEs [17]. Oh (2021) reported that non-heterosexuality was positively associated with PLEs among Latin Americans in the general population, though the association was not significant among Asian American [18]. To date, however, this link has not yet been reported in adolescents, a group with a high incidence of PLEs.

Sexual minority adolescents are more likely to feel unwelcome or unsafe in school due to unsupportive policies and stigma [19]. Bullying is a subset of aggressive behavior, which is generally defined as behavior intended to inflict injury or discomfort upon another individual [20]. Socially, sexual minority status is a risk factor for bullying on school property [21, 22]. In particular, Chinese sexual minority adolescents may perceive high levels of prejudice and rejection by their peers related to traditional Chinese social and cultural norms. A survey of Chinese high school students showed that the prevalence of school bullying experienced by gay and bisexual adolescents was as high as 25.9% and 16.2%, respectively, which was significantly higher than that of their heterosexual peers (12.1%) [23]. School bullying is a traumatic event that has been linked to increased adversity and mental health conditions [24]. For instance, a cross-sectional study of 623 high school students found that bullying in school led to the occurrence of PLEs [25]. Karcher and colleagues have found that children who endured a greater number of adverse childhood experiences, such as school bullying, experienced more PLEs [26]. While not all students experience bullying at school, the presence of bullying can make students feel threatened and powerless. Perceived school bullying can be understood as a subjective perception of bullying in schools, which directly reflects the safety of the school. One study has found that a strong association between perceived school safety and mental health problems in adolescents [27]. However, empirical data on the correlation between perceived school bullying and PLEs is lack.

Resilience generally refers to the adaptive ability to maintain an active life despite adversity and stressful event. In a compensatory model of resilience, it is acknowledged that there can be a direct positive effect of promotive factors on mental health outcomes, independent of the presence of risk factors [28]. The negative relationship between resilience and adolescent PLEs has been well elucidated [29, 30]. Meanwhile, previous work on bullying and mental health problems also has raised a host of modalities (i.e., mediating or moderating) through which resilience is related to mental health problems. The mediating and moderating role of resilience has been evidence in previous work with different hypotheses and analytical methods [31]. Specifically, resilience is a process of dynamic change whereby external protective factors from family, school, and peer groups can enhance individual resilience, while exposure to risky environments can diminish it [32]. Thus, resilience mediates the relationship between school bullying and poor mental health [33, 34]. On the other hand, resilience as an inherent personal attribute, it empowers individuals to thrive in the face of adversity [35]. The protective model of resilience supported that assets or resources moderate or reduce the effects of risks on a negative outcome [28]. In line with this model, some studies have showed that resilience can function as a moderator to buffer the negative effects of school bullying on mental health problems [36, 37].

Accordingly, resilience can play a mediating role by being negatively affected by adverse life events, such as perceived school bullying, which in turn can influence the development of mental disorders. On the other hand, resilience can also act as a moderate factor, providing protection against mental health issues. While some studies have shown that resilience can both mediate and moderate the relationship between childhood adversity and poor mental health [38, 39], these studies are limited by their cross-sectional designs, which make it difficult to establish causal inferences between variables. In this longitudinal study, sexual minority adolescents were surveyed in order to unpack the relationship linking perceived school bullying, resilience, and PLEs. Our major hypotheses are: (1) Perceived school bullying is positively significantly associated with PLEs in sexual minority adolescents; (2) Based on the dynamic process theory of resilience [32], resilience mediates the perceived school bullying-PLEs link; (3) According to the protective model of resilience [28], resilience moderates the perceived school bullying-PLEs link.

## Methods

### Participants and procedures

Data was extracted from the Mental Health Screening Program for Students in Bao’an district, Shenzhen (Guangdong province), China. This program aims to understand the mental health issues of primary and secondary school students in Bao’an district, Shenzhen in each semester through a convenience sample. Details of procedure can be found elsewhere [40]. In this study, only high school students were considered, so a total of 46 schools were included in the study. The data collection was conducted using the “Survey Star” system, which is a customized online platform developed specifically for this project by our team. Before each investigation, the local education bureau and target schools send the survey invitation letter to each participant and their guardians. Students and their guardians were required to agree and sign the informed consent form before participating in this study. Participants scanned the Quick Response (QR) code on their mobile phones to obtain an electronic version of the questionnaire to complete the survey. Student identities were anonymous across both surveys, and participants were permitted to interrupt or withdraw from the survey at any time. We also opened a psychological hotline (‘Xinqing’ hotline) to provide free help to students should they experience any psychological distress. After each survey, we provided training for all teachers to professionally respond to their students’ psychological needs. The investigation was carried out in accordance with the Helsinki Declaration as revised 1989 and approved by the Ethics Committees of South China Normal University (SCNU-PSY-2021-094).

**Fig. 1:**
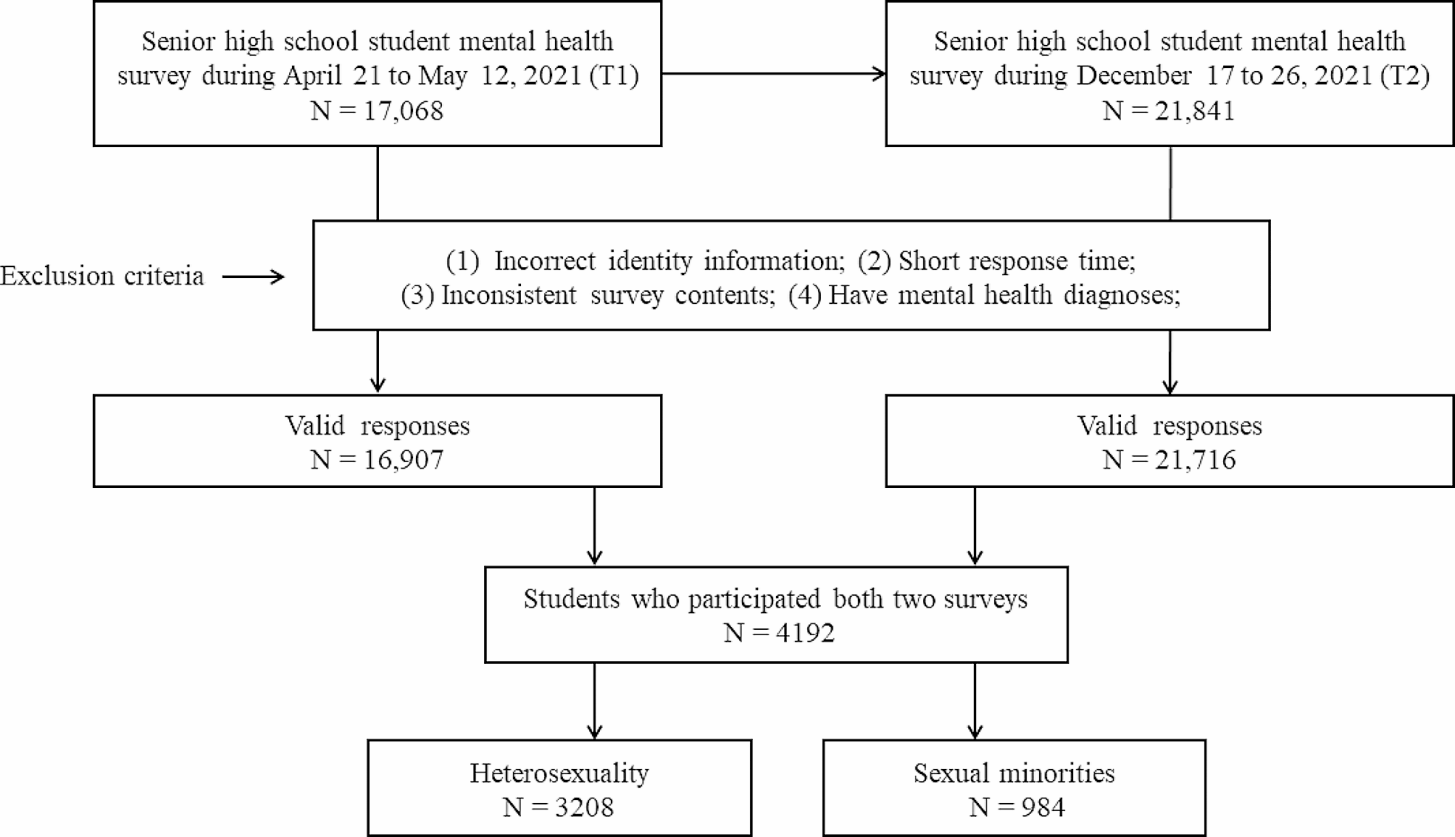
Participation flow chart

Two surveys were included in this study. As shown in Fig. 1, a total of 17,068 senior high school students (grades 10–12) were recruited in the first timepoint (Time1, T1: April 21 to May 12, 2021), and 21,841 students were recruited in the second timepoint (Time2, T2: December 17 to 26, 2021). To improve data quality, the exclusion criteria were as follows: (1) incorrect identity information, (2) short response time, (3) inconsistent survey contents, and/or (4) current mental health diagnoses that were either self-reported or reported by a teacher or guardian. A small number of participants met these exclusion criteria, leaving 16,907 and 21,716 students in the T1 and T2 sample, respectively. This study was a two-timepoint repeated cross-sectional survey with a nested longitudinal subsample. In T1 survey, students in the third year of senior high school (Grade 12) did not participate in T2 survey because they took the Chinese College Entrance Exam (i.e., Gaokao) in June 2021 and entered colleges/ universities. In T2 survey, the students of 10th graders did not participate in T1 survey, because they were still in junior high school when T1 survey started. Through data integration, a total of 4192 senior high students participated in all two web-based surveys and provided complete data on all measures. We used the χ^2^ test to compare the prevalence of T1 PLEs between participants who provided complete data across two waves and those who did not participate in the second survey (T2). There was no difference between these two groups (13.9% vs. 14.7%, χ^2^ = 1.35, *p* < 0.254). Among the respondents, a total of 984 students were sexual minorities (23.5%), including 19 gay men, 204 bisexual men, 22 lesbian women, and 739 bisexual women.

### Measures

#### Gender and sexual orientation

At T1, we obtained birth sex (male/female) of students from the local education bureau’s student status database. We reconfirmed participants’ gender orientation with one item (i.e., “What do you perceive your gender to be”) at T2. In this study, all participants fully identify with their biological sex (i.e., cisgender). Meanwhile, sexual orientation was used to evaluate by the single item Kinsey’s scale [41], which was back-translated into Chinese and has been validated in samples of Chinese adolescents [23]. Responses included 7 options: K0 = Exclusively heterosexual with no homosexual orientation; K1 = Predominantly heterosexual, only incidentally homosexual; K2 = Predominantly heterosexual, but more than incidentally homosexual; K3 = Equally heterosexual and homosexual; K4 = Predominantly homosexual, but more than incidentally heterosexual; K5 = Predominantly homosexual, only incidentally heterosexual; K6 = Exclusively homosexual. Respondents who chose mostly other sexual attraction (K1 and K2) and mostly same sexual attraction (K4 and K5) were categorized as having equally other and same sexual attraction (K3) [42]. To facilitate interpretation, we grouped the responses on the 7-point scale into three categories of sexual orientation: heterosexual (K0), homosexual (K6), and bisexual (from K1 to K5). Respondents with K1 to K6 scores were categorized as sexual minorities in this study.

#### Perceived school bullying

Participants’ perceived school bullying at T1 was assessed through the school bullying subscale, which derived from the 2016 Version of Delaware School Climate Scale-Student (DSCS-S) [43]. This subscale had four items, as follows: Item 9: Students threaten and bully others; Item 14: Students worry about others bullying them; Item 24: Bulling is a problem; and Item 27: Students bully one another. Responses were made on a four-point scale, ranging from 1 = strongly disagree to 4 = strongly agree. A higher total score reflected more perceived school bullying. The Chinese version of DSCS-S has demonstrated satisfactory psychometric properties [44]. In our study, the Cronbach’s α of the school bullying subscale was 0.72.

#### Resilience

Resilience was measured by the 10-item Connor-Davidson Resilience Scale (CD-RISC-10) [45] at T2. The item of CD-RISC-10 ranged from 0 = not true at all to 4 = true nearly all the time. A higher total score indicated greater resilience. The Chinese version of CD-RISC-10 has good reliability and validity [46, 47]. The CD-RISC-10 has been widely used in studies exploring the moderating [48, 49] or mediating role [50, 51] of resilience. The Cronbach’s α was 0.97 in this study.

#### PLEs

This study utilized the 8-item Positive Subscale of the Community Assessment of Psychic experiences (CAPE-P8) to measure current PLEs in both surveys. The CAPE-P8 originated in the Community Assessment of Psychic Experiences (CAPE) [52, 53]. There are 8 items in CAPE-P8, six of which assess delusional experiences (DEs), with the remaining items measuring hallucinatory experiences (HEs). Each item was rated within a time-frame of the last one month on a four-point Likert scale, from 1 = never to 4 = nearly always. Higher scores represent higher levels of PLEs. The CAPE-P8 has described acceptable psychometric properties in the Chinese adolescent sample [54, 55]. In the current study, Cronbach’s α scores were 0.89 and 0.91 at T1 and T2, respectively. Participants were categorized as having high frequent PLEs in the present study if they selected either “3-often” or “4-nearly always” or both items [9].

#### Covariates

The controlled sample characteristics include age, school type, ethnicity, parental marital status, family income, single child status, parental education, chronic physical illness status, and family history of psychiatric illness.

Negative life events experienced over the past six months was assessed at T2 by the Adolescent Self-Rating Life Events Check List (ASLEC) [56], which was consisted of 27 items, and clustered into six dimensions: interpersonal conflicts, academic pressure, being punished, personal loss, physical health problems, and others. Each was rated on a five-point Likert scale from 1 (not at all) to 5 (extremely severe), with higher total scores reflecting a higher level of stress. The Cronbach’s α of the ASLEC was 0.96 in this sample.

### Data analysis

We used SPSS 24.0 for all analyses. Descriptive statistics (Mann-Whitney U test and Chi-square test) were used to compare the sample characteristics and research variables between sexual minorities and heterosexual subjects. The Pearson correlation analysis was applied to examine the bivariate correlations between research variables. We tested for potential multicollinearity among all variables by variance inflation factor (VIF) [57]. We also conducted an exploratory factor analysis (EFA) to assess common method bias (CMB) [58]. The single factor explained by EFA did not explain more than 40% of the variance, indicating no significant CMB [59]. Furthermore, we tested the hypothetical mediation model using the PROCESS macro. We employed Model 4 to investigate the mediating role of resilience based on Hayes’ suggestions [60]. All continuous variables were standardized (converted to Z scores), which allows each variable to have the same range and variance. Such standardization procedures facilitate the comparison of results among different results. We entered T1 perceived school bullying as an independent variable, T2 resilience as the mediator, and T2 PLEs as the dependent variable. All demographic characteristics, baseline PLEs (T1), and negative life events (T2) were included as covariates in the analysis. In model analysis, the 5000 bootstrapping resamples method could provide 95% confidence intervals (95% CI) to estimate both direct and indirect effects simultaneously. Similarly, we explored the moderating effect of resilience using Model 1 [60]. We entered T1 perceived school bullying as an independent variable, T2 resilience as the moderator, T2 PLEs as the dependent variable, and all sample characteristics, baseline PLEs (T1), and negative life events (T2) as covariates. In addition, we have conducted separate analysis on the two dimensions (i.e., DEs and HEs) of PLEs as dependent variables to further examine if the results remain consistent across both dimensions.

## Results

### Descriptive statistics

The current study included 4192 senior high students, 984 of whom were sexual minorities (23.5%). Within the sexual minority sample, only 4.2% students (*N* = 41) reported they were homosexual, whereas 95.8% reported being bisexual (*N* = 943). The proportion of girls (22 lesbian women; 739 bisexual women) was much higher than boys (19 gay men; 204 bisexual men) (77.3% vs. 22.7%). The age of sexual minorities ranged from 14 to 19 years; the mean (SD) age was 16.68 (0.71) years. The vast majority of participants are of Han ethnicity and 22.3% were from single-child families. Other sample characteristics of sexual minorities are reported in Table 1.

**Table 1:** Sample characteristics of the participants (*N* = 4192)

Characteristics		Heterosexuality *N* = 3208	Sexual minorities *N* = 984	χ^2^ /Z	*p*-value
Age [year]	M(SD)	16.71(0.74)	16.68(0.71)	−1.14	0.254
Sex, [N(%)]	Boys	1761(54.9)	223(22.7)	313.81	< 0.001
	Girls	1447(45.1)	761(77.3)		
Grade, [N(%)]	10th	1947(60.7)	597(60.7)	0.00	1.00
	11th	1261(39.3)	387(39.3)		
School types, [N(%)]	Public schools	2157(67.2)	671(68.2)	0.31	0.586
	Private schools	1051(32.8)	313(31.8)		
Ethnicity, [N(%)]	Han^a^	3130(97.6)	956(97.2)	0.52	0.486
	Others	78(2.4)	28(2.8)		
Parental marital status	Good	2995(93.4)	887(90.1)	11.39	0.001
	Poor^b^	213(6.6)	97(9.9)		
Family income (monthly), [N(%)]	< 12000RMB	1268(39.5)	389(39.5)	0.68	0.878
	12,000 ~ 30,000 RMB	184(5.7)	61(6.2)		
	> 30,000 RMB	1039(32.4)	308(31.3)		
	Unknown	717(22.4)	226(23.0)		
Whether single child or not, [N(%)]	Yes	678(21.1)	219(22.3)	0.56	0.450
Paternal education, [N(%)]	Junior high school or below	1180(36.8)	352(35.8)	7.57	0.023
	Senior high school	115(34.8)	309(31.4)		
	College or above	913(28.5)	323(32.8)		
Maternal education, [N(%)]	Junior high school or below	1440(44.9)	424(43.1)	9.19	0.010
	Senior high school	11,033(32.2)	289(29.4)		
	College or above	735(22.9)	271(27.5)		
Chronic physical illness ^c^, [N(%)]	Yes	73(2.3)	47(4.8)	16.94	< 0.001
Family history of psychiatric illness, [N(%)]	Yes	17(0.5)	18(1.8)	15.36	< 0.001
T1 Perceived school bullying score	M(SD)	7.84(2.50)	8.23(2.23)	−4.83	< 0.001
T2 Resilience score	M(SD)	37.17(8.13)	34.10(7.70)	−10.90	< 0.001
T1 PLEs score	M(SD)	9.84(3.08)	11.06(3.61)	−12.20	< 0.001
T1 Frequent PLEs, [N(%)]	Yes	345(10.8)	238(24.3)	115.03	< 0.001
T2 PLEs score	M(SD)	9.88(3.13)	11.12(3.68)	−12.38	< 0.001
T2 Frequent PLEs, [N(%)]	Yes	297(9.3)	206(20.9)	97.24	< 0.001

^a^Han is the major ethnic group in China

^b^Poor parental marital status included separated, divorced, and widowed

^c^Chronic physical conditions referred to having at least one of arthritis, angina, asthma, diabetes, visual impairment, or hearing problem

The sample characteristics of the sexual minority group and of other adolescents are also shown in Table 1. The two groups were significantly different in terms of sex, parental marital status, father’s educations, mother’s educations, chronic physical illness, and family history of psychiatric illness. Details can be found in Table 1.

Table 1 also outlined that compared to their heterosexual peers, sexual minority students scored much higher on T1 perceived school bullying, T1 PLEs, and T2 PLEs, whereas they scored lower on T2 resilience. In addition, Sexual minority adolescents report more frequent PLEs at T1 and T2 than heterosexuality adolescents.

The VIF of T1 perceived school bullying, T2 resilience, and T2 PLEs were 1.09, 1.08, and 1.11, respectively, suggesting a low possibility of multicollinearity.

### Correlation analysis

T2 PLEs were positively associated with T1 perceived school bullying (*r* = 0.17, *p* < 0.001) while negatively associated with T2 resilience (*r* = −0.40, *p* < 0.001). T2 resilience had a negative relationship with T1 perceived school bullying (*r* = −0.19, *p* < 0.001).

### Mediation of resilience

An EFA found 12 factors with eigenvalues > 1 and the first factor accounted for 22.14% of the total variance, indicating that this sample is less affected by CMB. Table 2 depicts the standardized regression results of mediation to test the significance of the effects of T1 perceived school bullying on T2 PLEs through T2 resilience in sexual minority adolescents, after controlling for sample characteristics, baseline PLEs, and negative life events. T1 Perceived school bullying had a significant negative effect on T2 resilience (b = −0.12, *p* < 0.001). While T2 resilience negatively predicted T2 PLEs (b =−0.21, *p* < 0.001), the predictive effect of T1 perceived school bullying on T2 PLEs was not significant (b = −0.02, *p* = 0.434). Therefore, T2 resilience significantly and fully mediated the relation between T1 perceived school bullying and T2 PLEs in sexual minority adolescents (Model 1: b = 0.03, 95% CI = 0.01 ~ 0.04). The model explained 13.5% variances in T2 resilience and 37.7% variances in T2 PLEs. When separating different dimensions of PLEs, we found similar mediating roles of resilience in the relationship between perceived school bullying and DEs (Model 2: b = 0.03, 95% CI = 0.01 ~ 0.04) and HEs (Model 3: b = 0.02, 95% CI = 0.01 ~ 0.03).

**Table 2:** The meditation of resilience in the relationships between T1 perceived school bullying and T2 PLEs in sexual minority adolescents

	b	t	*p*	95%CI
*Model 1: T2 PLEs as an outcome variable*				
Perceived school bullying (T1)→Resilience (T2) (a)	−0.12	−3.79	< 0.001	−0.18, −0.06
Resilience (T2)→PLEs (T2) (b)	−0.21	−7.75	< 0.001	−0.26,− 0.16
Perceived school bullying (T1)→PLEs (T2)(c’)	−0.02	−0.78	0.434	−0.07, 0.03
Perceived school bullying (T1)→Resilience (T2)→PLEs (T2) (a*b)	−0.03	–	–	0.01, 0.04
*Model 2: T2 DEs as an outcome variable*				
Perceived school bullying (T1)→Resilience (T2) (a)	−0.12	−3.83	< 0.001	−0.18, −0.06
Resilience (T2)→DEs (T2) (b)	−0.23	−8.13	< 0.001	−0.28, −0.17
Perceived school bullying (T1)→DEs (T2)(c’)	−0.02	0.91	0.364	−0.08, 0.03
Perceived school bullying (T1)→Resilience (T2)→DEs (T2) (a*b)	0.03	–	–	0.01, 0.04
*Model 3: T2 HEs as an outcome variable*				
Perceived school bullying (T1)→Resilience (T2) (a)	−0.14	−4.42	< 0.001	−0.20, −0.08
Resilience (T2)→HEs (T2) (b)	−0.13	−4.21	< 0.001	−0.19, −0.07
Perceived school bullying (T1)→HEs (T2)(c’)	0.02	0.79	0.430	−0.04, 0.08
Perceived school bullying (T1)→Resilience (T2)→HEs (T2) (a*b)	0.02	–	–	0.01, 0.03

PLEs = psychotic-like experiences; DEs = delusional experiences; HEs = hallucinatory experiences; T1 = Time 1; T2 = Time 2

Model 1: adjusting for sample characteristics, T1 PLEs, and T2 negative life events

Model 2: adjusting for sample characteristics, T1 DEs, and T2 negative life events

Model 3: adjusting for sample characteristics, T1 HEs, and T2 negative life events

### Moderation of resilience

**Fig. 2:**
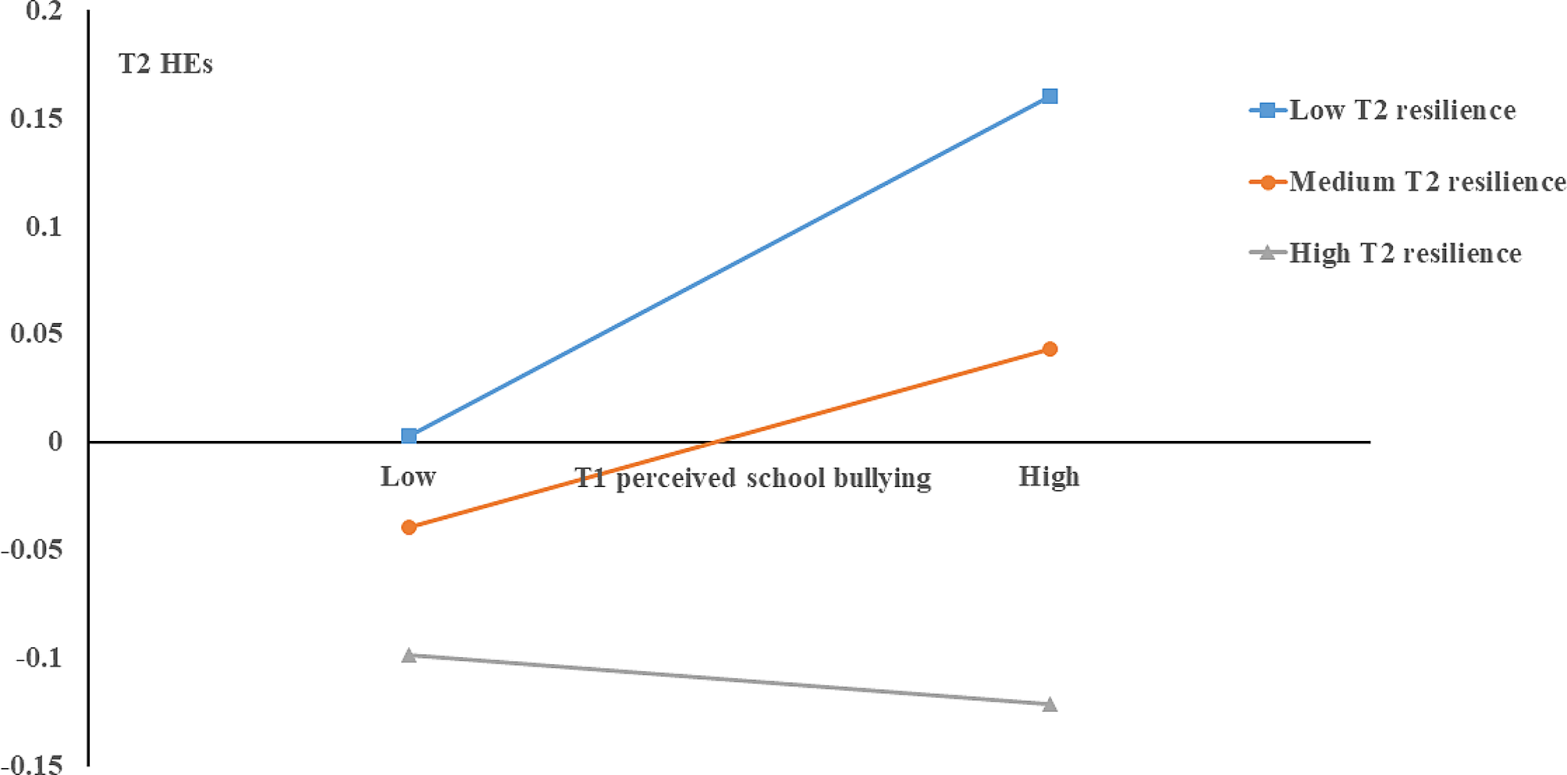
Interaction effect between perceived school bullying and resilience on hallucinatory experiences

The moderating effect was also tested, with T1 perceived school bullying entered as the predictor, T2 resilience as the moderator, and T2 PLEs as the outcome. Sample characteristics, baseline PLEs, and negative life events were included in the analyses as covariates. As shown in Table 3, T2 resilience had a main effect on T2 PLEs (b = −0.21, 95% CI = −0.26 ~ −0.16), while no significant effect of T1 perceived school bullying (b = −0.01, 95% CI = −0.07 ~ 0.04) and its interaction with T2 resilience (b = −0.04, 95% CI = -0.09 ~ 0.01) was found (Model 4). Thus, there was no identified moderation by resilience in the perceived school bullying-PLEs association in sexual minority adolescents. We further explored the moderating role of resilience between perceived school bullying and DEs (Model 5)/HEs (Model 6). Our data showed that the interaction between perceived school bullying and resilience did not significantly predict DEs (b = −0.04, 95% CI = −0.09 ~ 0.01), but significantly predicted HEs (b = −0.06, 95% CI = −0.12 ~ −0.01). Further, simple slope analyses found a significant positive relationship between perceived school bullying and HEs at low (b = 0.09, 95% CI = 0.01 ~ 0.17). However, when resilience reached a medium (b = 0.05, 95% CI = −0.02 ~ 0.11) or high level (b = −0.01, 95% CI = −0.08 ~ 0.05), there was no significant relationship between perceived school bullying and HEs. These findings indicated that a higher level of resilience may buffers the association between perceived school bullying and HEs (see Fig. 2).

**Table 3:** The moderation of resilience in the relationships between T1 perceived school bullying and T2 PLEs in sexual minority adolescents

	b	t	*p*	95% CI
*Model 4: T2 PLEs as an outcome variable*				
Perceived school bullying (T1)	−0.01	−0.44	0.656	−0.07, 0.04
Resilience (T2)	−0.21	−7.80	< 0.001	−0.26, −0.16
Perceived school bullying (T1) ×Resilience (T2)	−0.04	-1.78	0.076	−0.09, 0.01
*Model 5: T2 DEs as an outcome variable*				
Perceived school bullying (T1)	-0.02	−0.63	0.528	−0.07, 0.04
Resilience (T2)	−0.22	−8.18	< 0.001	−0.28, −0.17
Perceived school bullying (T1) ×Resilience (T2)	−0.04	−1.45	0.149	−0.09, 0.01
*Model 6: T2 HEs as an outcome variable*				
Perceived school bullying (T1)	0.04	1.21	0.225	−0.02, 0.10
Resilience (T2)	−0.23	−4.27	< 0.001	−0.19, −0.07
Perceived school bullying (T1) ×Resilience (T2)	−0.06	−2.28	0.023	−0.12, −0.01

PLEs = psychotic-like experiences; Des = delusional experiences; HEs = hallucinatory experiences; T1 = Time 1; T2 = Time 2

Model 4: adjusting for sample characteristics, T1 PLEs, and T2 negative life events

Model 5: adjusting for sample characteristics, T1 DEs, and T2 negative life events

Model 6: adjusting for sample characteristics, T1 HEs, and T2 negative life events

## Discussion

This longitudinal study aimed to advance our understanding of how perceived school bullying may be linked to PLEs by testing the mediating and moderating roles of resilience in a sample of sexual minority adolescents. Our findings showed that sexual minority adolescents perceived higher school bullying, reported lower resilience, and experienced more frequent PLEs than their heterosexual peers. Among sexual minority adolescents, resilience played a salient role in mediating this link, while the hypothesized moderation of resilience is not supported.

The current data showed that the prevalence of frequent PLEs was 24.3% at T1 and 20.9% at T2 for sexual minority adolescents, which is not only significantly higher their heterosexual peers (10.8% and 9.3%) in the current sample, but is also higher than one previous survey in Chinese junior and senior high school students using the consistent standard (15.4%) [9]. The Minority Stress Model [12] might explain this higher prevalence of PLEs among sexual minorities. The theory assumes that minorities experience consistently high levels of stress due to prejudice, discrimination, and/or stigma, which can lead to negative mental health outcome, such as PLEs. In addition, sexual minorities perceived higher bullying in school and reported lower resilience level than their heterosexual peers, an observation line with the existing literature [19, 61].

The cross-sectional association between school bullying and poor mental health has been firmly established in previous studies [24, 62]. As hypothesized, correlation analysis observed that perceived school bullying was positively associated with subsequent PLEs among sexual minority adolescents. However, the longitudinal significance did not remain after controlling for other variables in the regression model. This suggests that the longitudinal relationship between perceived school bullying and PLEs may be influenced by other additional variables. Among these potential variables, resilience is one of the most extensively studied factors [36, 37]. Adolescents with higher levels of resilience are less likely to experience PLEs [29, 30]. In the present study, the longitudinal association between perceived school bullying and PLEs was fully mediated by resilience. In other words, sexual minority adolescents with perceived school bullying might develop lower levels of resilience, rendering them more susceptible to stress, and thereby increasing the likelihood of experiencing PLEs in the future. The dynamic developmental view of resilience [32] supported that adolescents’ resilience is likely to be influenced by situational factors. We surmised that school bullying makes sexual minority adolescents aware of the potential risks posed by the school environment, thereby adversely affecting their resilience development and increasing their risk of PLEs. In light of these theories, we continued to test the mediating role of resilience when considering different types of PLEs in sexual minorities, and we found no significant differences. Specifically, we found that resilience fully mediates the relationship between school bullying and delusional experiences, as well as between school bullying and hallucinatory experiences.

Although some previous work observed the significant mediating but not moderating roles of resilience on other mental health problems, such as victimization-associated anxiety [34] and self-harm [33], fact that resilience is a buffer against negative stressful events is undeniable. The protection model also assumed that higher levels of resilience could moderate or decrease the adverse mental health effects via counter-acts and the acquisition of resources [28]. For instance, Schnarrs et al. proposed that resilience moderates the relationship between adverse childhood experiences and quality of physical and mental health in sexual minority adults [63], with a higher level of resilience, the negative effect of adverse childhood experiences on quality of physical and mental health would be diminished to a greater extent. However, we found only a moderating effect of resilience on the association between perceived school bullying and HEs, but limited moderating effect on the relationship between perceived school bullying and overall PLEs and DEs. This may reflect the different functions of DEs and HEs in adolescents and warrants further exploration in the future. In addition, sexual minority adolescents in the present study had impaired resilience compared to their heterosexual peers, which may partially explain the results of resilience fails to mitigate the impact of perceived school bullying on overall PLEs. Previous research has also pointed to the barriers sexual minorities face in accessing resources that can help increase resilience [61]. Impaired resilience among sexual minorities may hinder their ability to mobilize the resources (e.g., self-efficacy, family support) to buffer the impact of school bullying.

According to current findings, several implications were highlighted for sexual minority adolescents from an intervention and health-enhancement perspective. On the one hand, anti-school bullying interventions, such as civic education, cultural practices [64] and socio-environmental anti-bullying campaigns [65] may reduce bullying in schools. Also important are efforts to address those dysfunctions associated with poor resilience that can transform students’ mental wellbeing [66]. Finally, psychological therapies, such as cognitive-behavioral strategies, mentoring [67], mindfulness training, and psycho-social skills training [68] have all shown significant effectiveness in fostering resilience and promoting the development of mental health.

Finally, several limitations of this study should be noted. First, we used a self-reported scale to measure participants’ PLEs, which may have resulted in recall bias. In consideration of this possibility, it is worth noting that the false-positive rate of self-reports in the CAPE-P8, the measure for PLEs in the current study, has been shown to have acceptable self-evaluation-interview consistency (α = 0.78) in the Chinese population [55]. Second, we used only 4 items from the DSCS-S to examine school bullying. These items can only reflect the perceived bullying in the overall school climate, but cannot determine whether the participants suffered or participated in bullying. Thus, we could not tease out different types of bullying because of unavailable data. Meanwhile, we did not measure resilience at T1, so we are unable to account for its baseline level. Therefore, caution should be exercised when interpreting the current findings. Resilience can be regarded as either a trait or a state condition. Therefore, further research employing specific measurements for trait and state resilience is necessary to validate and corroborate our existing findings. Moreover, this exploratory study explored the relationship between perceived school bullying, resilience, and different PLE dimensions (i.e., DEs, HEs), but overlooked the potential influence of two components of PLEs on one another. In addition, there is a significant sex imbalance in sexual minorities (boys vs. girls: 22.7% vs. 77.3%), which may bias the current results. Consistent with previous studies showing the relevant preponderance of bisexual women compared to men [69], the number of bisexual women (*N* = 739) exceeded other sexual minority groups in our study. Lastly, sexual minorities in this study were screened from the total sample based on the Kinsey’s scale, a relatively old instrument that appears to screen a higher proportion of sexual minorities than in previous studies [10]. This high rate may be attributed to the inclusion of all adolescents who do not identify as heterosexual within the sexual minority group [42]. Senior high school students are still not fully mature in terms of sexual physiology and psychology, and thus may not yet be fully certain about their sexual orientation. In addition, differences in measures and sociocultural environments may also impact the detection of sexual minorities. Thus, the results need to be interpreted with caution.

## Conclusions

Our longitudinal study among Chinese sexual minority adolescents demonstrated that resilience mediates the association between perceived school bullying and PLEs, as well as moderates the association of perceived school bullying with HEs. These findings draw attention to the potential roles of educators and clinicians in considering and adopting measures to reduce school bullying and promote adolescents’ resilience. In light of our findings, further research will be needed to better understand the psychological mechanisms underpinning and linking school bullying, resilience, and PLEs in sexual minority adolescents.

## Data Availability

The dataset used and/or analyzed during the current study are available from the corresponding author (F.F) on reasonable request.
